# In vaccinated individuals serum bactericidal activity against B meningococci is abrogated by C5 inhibition but not by inhibition of the alternative complement pathway

**DOI:** 10.3389/fimmu.2023.1180833

**Published:** 2023-06-29

**Authors:** Emma Ispasanie, Lukas Muri, Marc Schmid, Anna Schubart, Christine Thorburn, Natasa Zamurovic, Thomas Holbro, Michael Kammüller, Gerd Pluschke

**Affiliations:** ^1^ Swiss Tropical and Public Health Institute, Molecular Immunology Unit, Basel, Switzerland; ^2^ University of Basel, Basel, Switzerland; ^3^ Novartis Institutes for Biomedical Research, Department Autoimmunity, Transplantation and Inflammation, Basel, Switzerland; ^4^ Novartis Pharma AG, London, United Kingdom; ^5^ Novartis Institutes for Biomedical Research, Translational Medicine-Preclinical Safety, Basel, Switzerland; ^6^ Global Drug Development, Novartis Pharma AG, Basel, Switzerland

**Keywords:** *Neisseria meningitidis*, serum bactericidal activity, complement inhibitors, serogroup B vaccine, alternative pathway, immunotherapy

## Abstract

**Introduction:**

Several diseases caused by the dysregulation of complement activation can be treated with inhibitors of the complement components C5 and/or C3. However, complement is required for serum bactericidal activity (SBA) against encapsulated Gram-negative bacteria. Therefore, C3 and C5 inhibition increases the risk of invasive disease, in particular by *Neisseria meningitidis.* As inhibitors against complement components other than C3 and C5 may carry a reduced risk of infection, we compared the effect of inhibitors targeting the terminal pathway (C5), the central complement component C3, the alternative pathway (FB and FD), and the lectin pathway (MASP-2) on SBA against serogroup B meningococci.

**Methods:**

Serum from adults was collected before and after vaccination with the meningococcal serogroup B vaccine 4CMenB and tested for meningococcal killing. Since the B capsular polysaccharide is structurally similar to certain human polysaccharides, 4CMenB was designed to elicit antibodies against meningococcal outer membrane proteins.

**Results:**

While only a few pre-vaccination sera showed SBA against the tested B meningococcal isolates, 4CMenB vaccination induced potent complement-activating IgG titers against isolates expressing a matching allele of the bacterial cell surface-exposed factor H-binding protein (fHbp). SBA triggered by these cell surface protein-specific antibodies was blocked by C5 and reduced by C3 inhibition, whereas alternative (factor B and D) and lectin (MASP-2) pathway inhibitors had no effect on the SBA of post-4CMenB vaccination sera.

**Discussion:**

Compared to the SBA triggered by A,C,W,Y capsule polysaccharide conjugate vaccination, SBA against B meningococci expressing a matching fHbp allele was remarkably resilient against the alternative pathway inhibition.

## 1 Introduction

Invasive meningococcal disease (IMD) is a potentially life-threatening infectious disease caused by the Gram-negative bacterium *Neisseria meningitidis.* Occasionally, it can become invasive and lead to meningitis and septicemia, as reviewed in (1, 2). A total of 6 of the 12 meningococcal capsular polysaccharides (A, B, C, W, Y, and X) account for most IMDs (3). In Europe, MenB is responsible for the majority of the IMD cases, as reviewed in (4, 5). Vaccines for meningococcal serogroups A, C, W, and Y are capsule polysaccharide-based, whereas vaccines against MenB are directed against outer membrane proteins. The MenB capsular polysaccharide is structurally similar to glycans of the human neural cell adhesion (6, 7) glycoprotein (N-CAM), and vaccination with this capsular polysaccharide would, therefore, carry a potential risk for autoimmune side effects. The two MenB vaccines currently available are 4CMenB (Bexsero, GlaxoSmithKline) and MenB-FHbp (Trumenba, Pfizer). 4CMenB is a multicomponent vaccine that contains three recombinant proteins that have been identified by reverse vaccinology approaches (5): neisserial heparin-binding antigen (NHBA), neisserial adhesin A (NadA), and variant 1 of the factor H-binding protein (fHbp), along with outer membrane vesicles (OMV) (8, 9). The four main alleles of NadA, which mediates bacterial cell adhesion and invasion, are distinguished (10). The highly polymorphic lipoprotein NHBA increases serum resistance by binding to heparin through arginine-rich regions. FHbp, a surface-exposed lipoprotein, is a key meningococcal virulence factor that has evolved to escape complement-mediated attack by binding to human complement factor H (FH) and recruiting it to the bacterial cell surface (11–14). FH is the major negative regulator of the alternative complement pathway (AP) that dissociates the C3 convertase and acts as a cofactor for factor I to cleave and inactivate C3b. The AP acts as an amplification loop for both the CP and the LP where C3b binds to factor B (FB), which is then cleaved by factor D (FD) to form the AP C3 convertase C3bBb (15, 16). The activation of the AP could also be mediated by the spontaneous hydrolysis of C3, resulting in the formation of C3(H_2_O), a process called the “tickover” of C3. The change in shape allows the binding of plasma protein FB, which then also undergoes a conformational change that renders it susceptible to the recruitment and cleavage by protease FD into Ba and Bb. The Bb protein remains bound to C3(H_2_O), resulting in the formation of fluid-phase C3 convertase C3(H_2_O)Bb. This C3 convertase has its own serine protease that can cleave C3 to yield C3a and C3b (17–19). This C3b, although formed in small amounts, can attach covalently to the surface of pathogens. The bound C3b is able to bind FB, which is then cleaved by FD, to yield small fragment Ba and the active protease Bb. The AP C3 convertase C3bBb is stabilized by properdin and prolongs its half-life, enabling further C3 cleavage and amplification of complement activation. Additional C3b can bind to an existing C3 convertase and form a C5 convertase, C3b_2_Bb. The C5 convertase cleaves C5 to C5a and C5b, the latter of which is the catalyst for the membrane attack complex (MAC) formation, which is the most potent human weapon against meningococci. By recruiting FH to the cell surface, fHbp can, therefore, reduce AP activation and MAC formation on the bacterial surface and thereby prevent bacterial killing (20). Most invasive meningococcal isolates independent of serogroup harbor one of three main variants (v. 1, 2, or 3) of the *fhbp* gene in the genome (21). Serum antibodies to fHbp in the variant 1 group have been shown to be bactericidal against isolates with variant 1 proteins, but they lack cross-protection towards isolates with variant 2 and 3 proteins. Antibodies to fHbp in the variant 2 or 3 groups are mainly active against isolates with homologous variants, i.e., two or three proteins (21–23). Assessing the immunogenicity of each vaccine component in 4CMenB has been a major challenge. Several typing systems such as MATS (Meningococcal Antigen Typing System) have been used to predict the coverage afforded by 4CMenB. The MATS sandwich ELISA uses bacterial lysates and rabbit antisera to the target antigens to correlate the susceptibility of serogroup B strains to SBA (9, 20, 24). This has demonstrated that removal of anti-fHbp antibodies from the serum of 4CMenB-immunized adults reduced SBA by 88 to >95 percent (25), suggesting that antibodies targeting fHbp may be crucial for successful protection.

As detailed and reviewed in Lewis and Ram (2020), the complement system and, in particular, the formation of the membrane attack complex C5b-9 present the major defense mechanism against meningococcal infections (26). Vaccination with either a polysaccharide-based or MenB vaccine has been shown to induce serum bactericidal antibodies, which are able to activate the classical pathway (CP) that leads to MAC formation and bacteriolysis (15). As reviewed in Rawal and Pangburn (2003), antibody-mediated activation of the CP starts with the binding of the C1 complex to the Fc region of the antibodies that are bound to their target antigen on the bacterial cell surface. C4 gets recruited to this complex and is cleaved into C4b, which becomes covalently deposited on the meningococcal cell surface. C4b recruits C2 that is cleaved by C1s into C2a and C2b, and the C2a part is bound to C4b (18). The lectin pathway (LP) is initiated by binding of the carbohydrate recognition molecules mannan-binding lectin (MBL), ficolins, and collectin 11 to the carbohydrate motives on the surface of microbes and cells, followed by the activation of MASP-2 (an essential enzyme responsible for the LP activation) that cleaves both C4 and C2 to create the C3 convertase C4b2a (27). The C4b2a complex is a C3 convertase that cleaves C3 into C3a and C3b, and C3b binds to C4b2a to form a C5 convertase (C4b2a3b). The C5 convertase then cleaves C5 into anaphylatoxin C5a and C5b, and C5b can interact with C6 to form C5b6. This complex is then able to immediately bind to C7, which exposes a hydrophobic domain that allows the C5b-7 complex to bind to the bacterial membrane. Subsequently, recruitment of C8 results in the first membrane protrusions, and further recruitment of several C9 proteins forms the MAC pore that ruptures the membrane and lyses the meningococcal cell (17, 18).

Inhibitors of complement factor C5 and, more recently, C3 are approved for treating several diseases caused by dysregulation of the complement system. However, C5 inhibition is associated with increased risk of meningococcal infections, while this has so far not been confirmed for C3 inhibitors. As potential alternatives, AP inhibitors are being profiled by several companies. In a previous study, we found that successful vaccination with the meningococcal polysaccharide vaccine MenACWY-CRM_197_ (Menveo^®^) reduces the requirement of the amplification loop for meningococcal SBA (28). Here, we analyzed whether vaccination with the protein-based 4CMenB vaccine has a similar effect and tested the impact of complement inhibitors targeting the terminal pathway (C5), C3, the AP (FB, FD), and the LP (MASP-2) on *N. meningitidis* SBA against serogroup B isolates by serum collected pre- and post-vaccination with the 4CMenB vaccine.

## 2 Methods

### 2.1 Ethical approval

The experiments involving human specimens were approved by the Ethical Committee of Northwest and Central Switzerland (Ethikkommission Nordwest- und Zentralschweiz (EKNZ), Studie 2018-02341).

### 2.2 Bacterial isolates

*Neisseria meningitidis* serogroup B isolate 6105 was obtained from the Institute for Infectious Diseases (University of Bern), isolate 4263 from Mark Achtmann (MPI for Infection Biology, Berlin), and isolate MC58 from ATCC (**Table 1**).

**Table 1:** Serogroup B *Neisseria meningitidis* isolates.

Isolate	Origin	Year of isolation	MLST*	Source	PorA VR	fHbp variant (v), peptide ID	NadA allele
6105	Switzerland	2016	11063	case	19,15	v.1 ID1	2/3
4263	USA	1994	11	case	5,2	v.3 ID98	2/3
MC58	UK	1985	74	case	1.7,16b	v.1 ID1	1

All strains were characterized for their MLST and their *porA, fHbp*, and *nadA alleles* by PCR amplification and sequencing. *MLST, multi-locus sequence typing; fHbp, factor H-binding protein; PorA, Porin A; VR, variable regions; NadA, neisserial adhesin A.

### 2.3 DNA sequencing of the *fHbp* and *nadA* genes

Sequencing of the *fHbp* and *nadA* genes was conducted as previously described. The isolates were sub-cultured on chocolate agar plates (Biomerieux) overnight at 37°C and 5% CO_2_. Mid-log phase bacteria grown in a Frantz medium (28) were pelleted, and genomic DNA was extracted using the QIAmp DNA Mini Kit (Qiagen) according to the manufacturer’s instructions. The *fHbp* and *NadA* genes were PCR amplified using primers as described in Ispasanie et al. (2014) (29). The same primers were also used for sequencing. The sequences were analyzed with MEGA-X v.10.1.8, and the fHbp amino acid sequence identification number (variant, peptide ID) and NadA allele were identified and designated using the online Neisseria Sequence Typing database (http://pubmlst.org/neisseria).

### 2.4 Human sera

Four healthy volunteers were recruited upon informed and written consent (**Table 2**). All had been immunized with meningococcal capsule polysaccharide vaccines [MenACWY-CRM_197_ (Menveo^®^)], NeisVac-C (Pfizer), Mencevax™ (Pfizer), and unconjugated MenA+C (Sanofi-Pasteur) prior to enrolment. Three subjects, 3, 11, and 12, received two doses of the meningococcal protein vaccine 4CMenB in 2019 in the framework of this study. The second dose was given 2 months after the first one. Subject 2, who had been immunized with the recommended two doses of 4CMenB in 2016, only received one booster shot in 2019. Venous blood was taken before (Pre), 2 months post-dose 1 (mPD 1), 2 weeks post-dose 2 (wPD 2), and 6 months post-dose 2 (6mPD 2). For subject 2, blood was taken before (Pre), 2 weeks (wPB), 2 months (mPB), and 6 months post-booster (6mPB). Vacuette^®^ CAT serum tubes (Greiner Bio-one) were used to prepare the sera, and preservation of the terminal complement activity was reconfirmed (Complement TCC, Svar).

**Table 2:** Study subjects.

Subject number	Age	Sex	4CMenB vaccinations (month, year)
2	31	F	03.2016, 05.2016, **boost**: 09.2019
3	66	M	09.2019, 11.2019
11	43	F	09.2019, 11.2019
12	29	M	09.2019, 11.2019

Subject numbers of this study are not consecutive. The volunteers were part of a cohort receiving different vaccines, and the subjects included in this study include all subjects receiving the 4CMenB vaccine and do not reflect a selection. M, male; F, female.

### 2.5 Determination of protein-specific IgG titers

Serum anti-fHbp IgG titers were measured by ELISA as previously described (30). Maxisorp 96-well plates (Nunc) were coated overnight at 4°C with 2 μg/mL of non-lipidated recombinant hexa-histidine-tagged fHbp variant 1 ID1 and NadA variant 2/3 in PBS (10). Serum samples were serially diluted five-fold, starting at 1:25 in blocking buffer (PBS + 2% milk). After serum incubation for 2 hours at room temperature (RT), the plates were incubated for 1 hour at RT with secondary antibody HRP-conjugated goat-anti human IgG H+L (1:3000 dilution, Bio-Rad). The titer was defined as the extrapolated dilution resulting in absorption of 1 at 450 nm following the addition of KPL peroxidase substrate (SeraCare) and 0.5M sulfuric acid.

### 2.6 SBA assay

Serum bactericidal titers were measured as described previously (28). Approximately 400 CFUs of bacteria were grown in a Frantz medium containing 2mM cytidine-5′-monophospho-N-acetylneuraminic acid (CMP-NANA, Sigma-Aldrich) and 20% human serum with internal complement preserved to the mid-log phase. The interpolated dilution of sera resulting in the 50% killing of bacteria after 60-minutes incubation at 37°C compared to the control reactions, without active serum and at time 0, was determined. Antibody titers were log10 transformed using GraphPad Prism v.8.2.1, and concentrations of <5 were assigned the value 2 (100% survival). To investigate the contribution of different complement components during immunity against serogroup B meningococci, selected complement inhibitors of the terminal, alternative, and lectin pathway were used. An anti-C5 antibody [tesidolumab, LFG316, Novartis (31, 32)] and an anti-MASP-2 antibody (produced in-house with the same sequence and properties as narsoplimab) (33, 34) together with factor B (iptacopan, LNP023, Novartis (35),), factor D (CMS487, Novartis (35),), and C3 (CP-40, Bachem) inhibitors were diluted in *Dulbecco’s* phosphate-buffered saline (DPBS) and added to the SBA reaction mixture. After incubation at 37°C for 60 minutes, the percent survival was assessed by plating on a chocolate agar PolyViteX (Biomerieux) and compared to the CFUs in the control reactions at time 0.

### 2.7 Statistical analysis

Pearson’s correlation was used to determine the correlation between SBA and serum anti-fHbp and anti-NadA IgG concentration. The effect of inhibitors on the survival of the serogroup B isolates in the post- and pre-vaccination sera was evaluated using a two-way ANOVA and Tukey’s multiple comparison test.

## 3 Results

### 3.1 Anti-fHbp IgG titers elicited by 4CMenB vaccination correlate with SBA

To characterize the antibody response to vaccination with 4CMenB, antibody titers against fHBp v.1 and NadA v.2/3 were determined pre- and post-vaccination by ELISA. Sera from four healthy individuals, including three vaccination-naïve subjects and one subject that had received two doses of 4CMenB three years prior to this study, were collected prior to vaccination and at different time intervals after vaccination. IgG titers specific to the vaccine antigen allele fHbp v.1 ID1 and NadA v.2/3 were determined by ELISA (**Table 3**).

**Table 3:** Summary of the anti-fHbp v.1 ID1 and anti-NadA v.2/3 IgG antibody titers in serum samples from subjects vaccinated with 4CMenB.

Subjects	2 (pre-vaccinated)				3 (vaccine-naïve)				11 (vaccine-naïve)				12 (vaccine-naïve)			
Serum sample	Pre	wPB	mPB	6mPB	Pre	mPD 1	wPD 2	6mPD 2	Pre	mPD 1	wPD 2	6mPD 2	Pre	mPD 1	wPD 2	6mPD 2
Anti-fHbp v.1 ID1 titers	321	15370	16469	8318	160	37440	37440	18909	20	5050	2417	1862	20	2951	575	575
Anti-NadA v.2/3 titers	96	6658	4935	3043	34	27	60	86	20	2590	26	148	20	1953	89	163

Serum IgG titers were determined based on the serum dilution calculated to give an OD450 intercept of 1. A titer of 20 (below the lowest serum dilution tested) was given when the curve did not intercept at 1. pre-vaccination serum sample (Pre), 2 weeks post-booster (wPB), 2 months post-booster (mPB), 6 months post-booster (6mPB), 2 months post-dose 1 (mPD 1), 2 weeks post-dose 2 (wPD 2), and 6 months post-dose 2 (6mPD 2).

The previously immunized subject 2, and one of the three vaccine-naïve subjects (subject 3), had measurable anti-fHbp IgG titers (321 and 160, respectively) prior to 4CMenB vaccination (**Table 3**). All subjects showed marked anti-fHbp IgG responses with titers between 2951 and 37440 after the first immunization (mPB or mPD 1), which did not increase further in response to the second immunization in the vaccine-naïve subjects. For unclear reasons, titers even declined for subjects 11 and 12 (**Table 3**). Six months after the boost or the second immunization (6mPB and 6mPD2), titers had declined to 19 -54%.

To confirm the key role of anti-fHbp antibodies in complement-mediated bacterial lysis, SBA titers against three meningococcal B isolates were determined. Strain 6105 expressed both fHbp v.1 ID1 allele and NadA 2/3 allele included in the vaccine, whereas the other strains expressed either the matching fHbp v.1 ID1 allele and a different NadA allele (strain MC58) or the matching NadA 2/3 allele and a different fHbp allele (strain 4263). Through the 4CMenB vaccinations, sera with a broad spectrum of IgG titers against fHbp variant 1 (between 20 and 37440) from the four sampling time points thus became available for analyzing the SBA.

**Figure 1:**
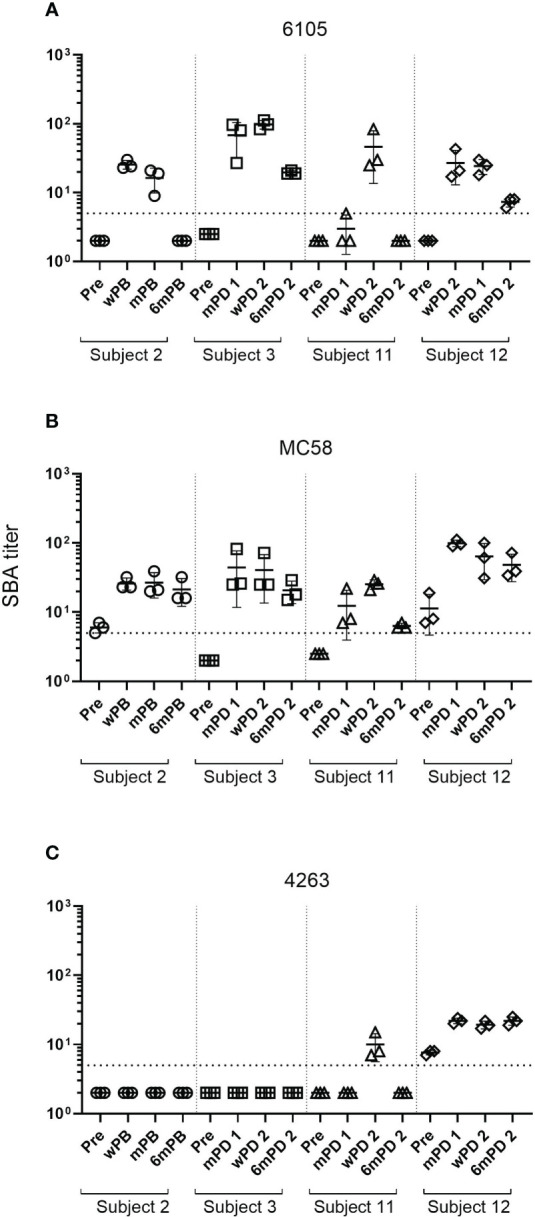
Changes in SBA titers against meningococcal B isolates after 4CMenB vaccination. SBA titers of sera from subject 2 collected prior to the 4CMenB booster vaccination (Pre), 2 weeks (wPB), 2 months (mPB), and 6 months post-booster (6mPB) and from subjects 3, 11, and 12 collected before vaccination (Pre), 2 months post-dose 1 (mPD 1), 2 weeks post-dose 2 (wPD 2), and 6 months post-dose 2 (6mPD 2) were determined against the serogroup B isolates 6105 **(A)**, MC58 **(B)**, and 4263 **(C)** Bactericidal titers are the reciprocal dilutions of serum that resulted in 50% killing of the bacteria after 60 minutes incubation. Error bars represent standard deviation of the mean titer of triplicate technical replicates. The bottom dotted line indicates the lowest serum dilution measured (1:5). Symbols below that line are set to 2 to indicate SBA titers below the detection limit. Serum bactericidal antibodies were induced in all subjects after the first immunization for isolates 6105 and MC58 but only for subject 12 for isolate 4263. Antibody titers declined for all subjects six months post-dose 2 for isolates 6105 and MC58 and subject 11 for isolate 4263. Titers for subject 12 measured against isolate 4263 remained high.

**Figure 2:**
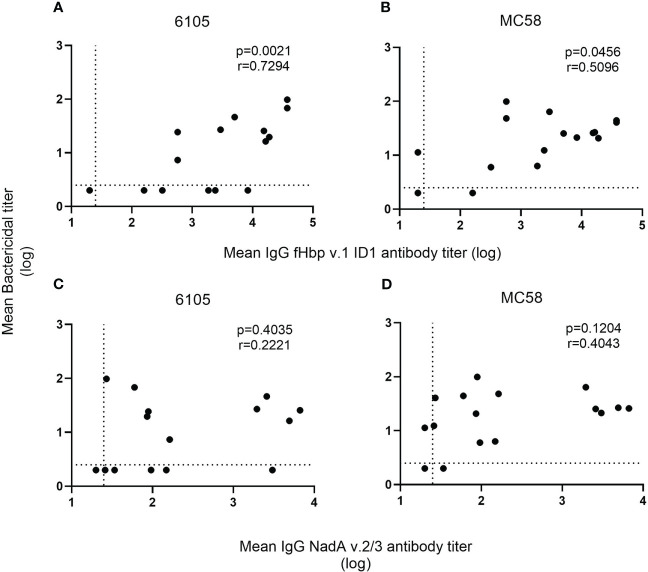
Correlation of SBA and anti-fHbp v.1 1D1 and NadA v.2/3 IgG titers. For each recombinant protein, the mean concentration of anti-fHbp v.1 1D1 **(A, B)** and NadA v.2/3 IgG **(C, D)** was plotted against the corresponding mean SBA titer against the serogroup B isolates 6105 **(A, C)** and MC58 **(B, D)**. The dashed horizontal line specifies the SBA titer detection limit (2.5), and symbols set below that line indicate a negative SBA (2). While the dashed vertical line shows the lowest serum dilution tested (1:25), symbols set below that line indicate an antibody titer with an intercept of less than 1 at OD450 (20). The correlation between the two non-parametric paired datasets were calculated using GraphPad Prism v.8.2.1 and the Spearman’s correlation coefficient test (r). *P*-values of ≤0.05 were considered to be statistically significant. Analyses show that an increase in SBA is correlated with an increase in anti-fHbp serum bactericidal antibodies but not with anti-NadA antibodies.

Despite the low anti-fHbp antibody titers (321 and 160), no SBA activity was observed in the pre-vaccination sera of donors 2 and 3. 4CMenB immunization led to an increase in SBA titer in all four study subjects against both strains 6105 and MC58 with matching fHbp allele (**Figure 1**, **Supplementary Table 1**). SBA titers increased after the first immunization and remained high after the second. For the subject 11/strain 6105 combination, an increase in SBA titer was seen only after the second immunization (**Figure 1A**), although IgG titers against fHbp already showed an increase after the first immunization (**Table 3**). SBA titers dropped drastically at 6 months (**Figure 1A**) for strain 6105 and less pronouncedly for strain MC58. Anti-fHbp IgG and SBA titers showed a statistically significant positive correlation for both fHbp v.1 ID1 allele-expressing strains (**Figures 2A, B**).

SBA activity was much more limited against strain 4263, which expresses a matched NadA but a heterologous fHbp allele. Despite the high ELISA antibody titers against NadA, subjects 2 and 3 developed no SBA against strain 4263, while subject 11 showed a transient and limited SBA at only two weeks after the second immunization (**Supplementary Table 1**). In contrast, subject 12 had some pre-vaccination SBA against strain 4263, which was enhanced by vaccination (**Figure 1C**) and may be related to antibodies specific to surface-exposed antigens of the OMV component of 4CMenB (36).

In contrast to the anti-fHbp IgG titer, there was no correlation between SBA and the anti-NadA IgG titer (**Figures 2C, D**). In subjects 2 and 3, low IgG titers specific to NadA v.2/3 prior to vaccination (96 and 34, respectively) were found. The pre-vaccinated subject 2 showed a marked titer increase to 6658 after the booster immunization, and titers remained high (>3000) for 6 months (**Table 3**). Marked titer increases (2590 and 1953, respectively) were also elicited in the vaccine-naïve subjects 11 and 12 in response to the first immunization, but the titers declined (<200) after the second immunization. Only minor anti-NadA IgG titer increases (<100) were observed for the vaccine-naïve subject 3. Taken together, the data confirm that antibodies against fHbp are critical for SBA against serogroup B meningococci and that antibodies to NadA v.2/3 provide limited protection.

### 3.2 Effect of complement inhibitors on SBA

**Figure 3:**
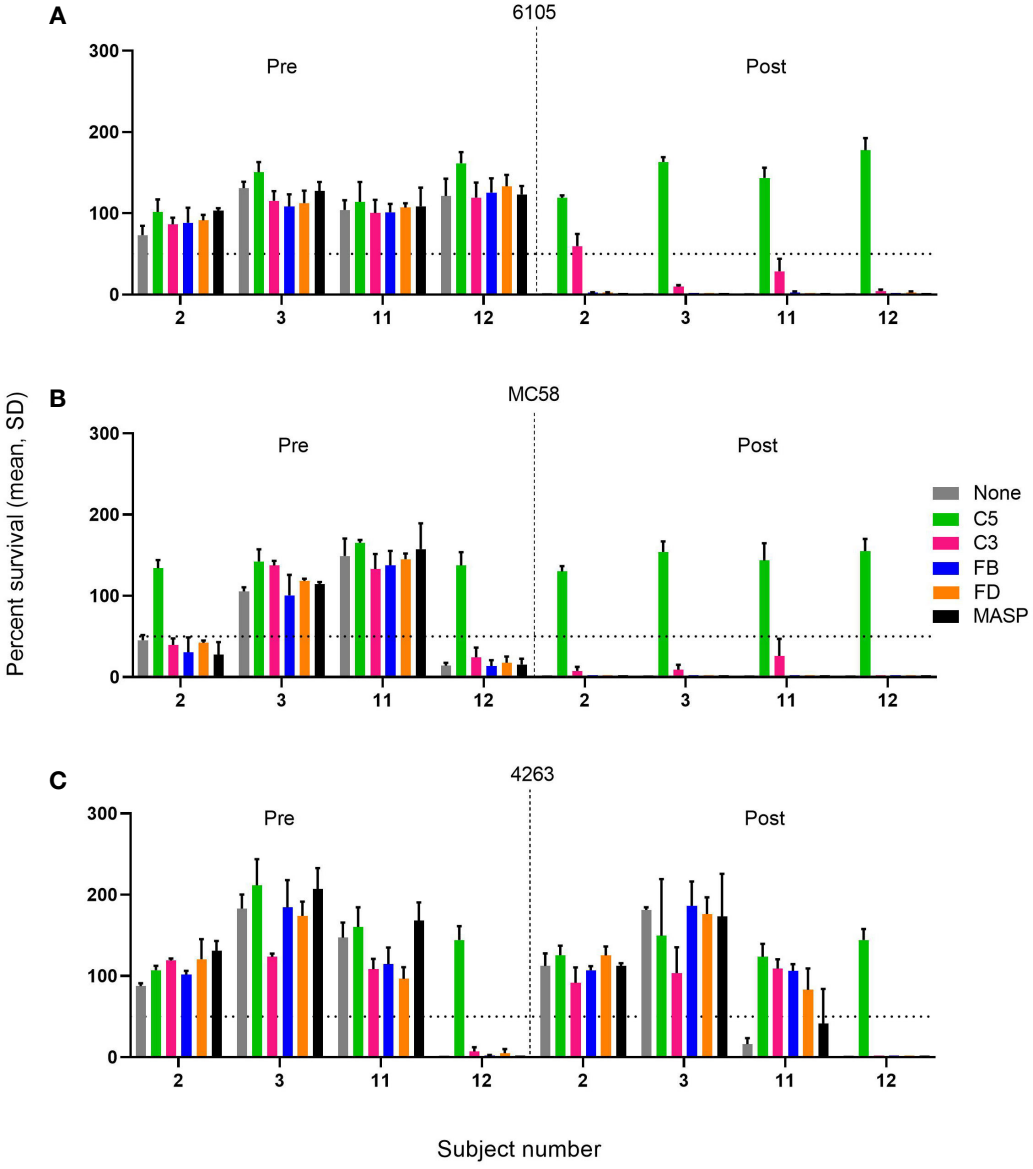
Effect of C5, C3, AP, and LP inhibitors on the survival of meningococcal serogroup B isolates in sera taken prior to vaccination (Pre) or 2 weeks after the boost of the pre-vaccinated individual or the second vaccination for the vaccine-naïve subjects (Post). Sera were diluted 1:5 and assayed with the internal complement. Data shown in the graphs are with the highest inhibitor concentration tested: FB: 25 µM, FD: 25 µM, C3, anti-C5, and anti-MASP-2 mAb: 100 µg/mL for the three serogroup B isolates 6105 **(A)**, MC58 **(B)**, and 4263 **(C)**. The horizontal dotted line represents 50% survival of bacteria after 60 minutes incubation as compared to the control wells at time 0. Data for each inhibitor are triplicate technical replicates. SD: standard deviation. Vaccination with 4CMenB reduces the effect of complement inhibitors FB, FD, and C3 on the SBA killing of the meningococcal isolates 6105 and MC58.

The effect of complement inhibitors on the killing of the meningococcal strains was established at inhibitor concentrations of 1-100 µg/mL for the anti-C5 mAb tesidolumab, the anti-MASP-2 mAb, and the C3 inhibitor CP-40 and 0.05-25 µM for the FB inhibitor iptacopan and FD inhibitor CMS487 (**Supplementary Figure 1**). As is typical, full inhibitory activity was already reached at concentrations of 50 µg/mL for the C3 and C5 inhibitors. Representative results for the C3, FB, FD, C5, and MASP-2 inhibitors at the highest concentrations tested are shown in **Figure 3** for sera taken before vaccination or 2 weeks after the boost of the pre-vaccinated individual or the second vaccination of the vaccine-naïve subjects.

In pre-vaccination sera without marked SBA activity, the presence of either of the complement inhibitors had a limited effect on bacterial growth compared to the controls. However, in the case of the three pre-vaccination serum/strain combinations with significant SBA (2/MC58, 12/MC58, and 12/4263), C5 inhibition reduced the bactericidal activity substantially, whereas this was not observed for any of the other complement inhibitors. In these sera, SBA may be triggered by antibodies directed against a variety of cell surface antigens elicited by natural exposure. The SBA of the post-vaccination sera against strains 6105 and MC58 (**Figures 3A, B**), which seems to be triggered primarily by anti-fHbp v.1 ID1 antibodies, was also abrogated by C5 inhibition. In addition, C3 inhibition caused a variable but limited reduction of SBA, whereas inhibitors of the AP or LP did not impact SBA. The effect of the C3 inhibitor CP-40 did not increase when the concentration was increased to 1 mg/mL. A similar inhibition of SBA was also caused by the anti-C5 antibody in subject 12 with pronounced bactericidal activity against strain 4263. Intriguingly, the weak and transient SBA that was observed in subject 11 in serum taken 2 weeks after the booster vaccination showed more pronounced dependency on the AP, suggesting that at limited SBA titers, the amplification loop may be critical for bacterial clearance.

## 4 Discussion

A well-established treatment option for paroxysmal nocturnal hemoglobinuria (PNH) and atypical hemolytic uremic syndrome (aHUS) caused by dysregulation of complement activation is based on the prevention of MAC formation with eculizumab. This mAb prevents cleavage of C5 to C5a and C5b; this is further reviewed in (16). However, MAC formation is required for SBA against Gram-negative bacteria, in particular by *Neisseria meningitidis*, and compared to the general population, eculizumab immunotherapy increases the risk of meningococcal disease approximately 2000-fold (37–39). Therefore, it is recommended that individuals receiving eculizumab are vaccinated with a capsule-conjugate vaccine against non-B meningococci and with a MenB protein vaccine. Recently, the C3 inhibitor pegcetacoplan has also been approved for the treatment of PNH (40), and new additional therapies targeting other pathways of the complement system to mitigate safety concerns are being developed. We previously investigated the survival of serogroup A, C, W, and Y meningococci in serum from individuals pre- and post-vaccination with the conjugate MenACWY vaccine Menveo and assessed the effect of inhibitors of FB, FD, C3, C5, and MASP-2 (28). Our data showed that the AP inhibitors FB and FD did not impair the killing of meningococci in sera from individuals with high bactericidal anti-capsular IgG titers and had minor effects in sera with moderate titers.

The two MenB vaccines 4CMenB and MenB-FHbp both include fHbp, which is harbored by nearly all meningococcal lineages and is a highly immunogenic target of bactericidal antibodies elicited by vaccination (41). However, fHbp antibodies provide only limited cross-protection between sub-families. Both the dominating potent bactericidal activity of anti-fHbp antibodies and their limited cross-protection were reconfirmed in our study. We found that after 4CMenB vaccination, the sera had highly effective SBA against MenB isolates expressing a matching fHbp allele. While SBA triggered by the anti-fHbp antibodies was blocked by C5 and reduced by C3 inhibition, the AP inhibitors had no effect. The limited effect of C3 inhibition on SBA is indicative of a bypass pathway for C5 activation. While C3b is a key component of both CP and AP C5 convertases, it has been demonstrated that a complex formed by a covalent dimer of C4b and by C2a can activate the transformation of C5 into C5a and C5b (42). This C3-bypass pathway may be responsible for the observed induction of SBA in the presence of the C3 inhibitor CP-40.

SBA is triggered by antibodies specific to antigens that are highly expressed on the bacterial cell surface. However, the threshold density of antigen–antibody complexes required for complement activation can also be reached by the simultaneous binding of antibodies to multiple surface-exposed antigens (43). While this may be relevant for an enhancing effect of the OMV component of 4CMenB, as reviewed in (36), it may also be responsible for natural immune responses, such as the pre-vaccination SBA against strains MC58 and 4263 found for subject 12. In spite of the high anti-NadA IgG titers, SBA activity against strain 4263 expressed the matched NadA but a heterologous fHbp allele that was very limited, confirming that the fHbp component of 4CMenB is more efficacious than NadA.

Our data indicate that the anti-fHbp antibodies drive MAC formation *via* the classical pathway very efficiently and are largely independent on the AP-mediated amplification loop. Likewise, Konar and Granoff (44) observed that meningococcal killing in whole blood from individuals immunized with 4CMenB was much less impaired by the FD inhibitor ACH-4471 than by eculizumab. In a previous study, we observed that pre-vaccination sera may have AP-dependent SBA against non-serogroup B meningococci (28). In contrast, AP downregulation by the α(2–8)-linked sialic acid capsule homopolymer of B meningococci (45) is making MenB isolates widely resistant to AP-mediated killing. Experiments with strains differing in fHbp expression levels have shown that dependence on the AP may increase at low epitope density (46). In agreement with a systematic review and meta-analysis for randomized clinical trials with 4CMenB (47), we observed a strong increase in SBA after the primary course of 4CMenB and only a moderate enhancement by the booster immunization. Six months after the booster immunization, antibody titers had declined substantially in our study, indicating a need for booster immunizations to keep antibody levels high. In a study of the longer term persistence of SBA in adolescents and young adults, robust immunological responses were observed following booster vaccinations at 4 years and 7.5 years after primary vaccination (48).

Related to ethical considerations, the limitations of the present study included the use of sera from adults rather than from children and the limited number of volunteers included. However, through the two-course vaccination, sera with a broad spectrum of IgG titers against fHbp became available from the four sampling time points.

The LP is the third pathway in the complement system, and it is initiated by the binding of mannose-binding lectin (MBL) to certain sugars on the bacterial surface. The multimers of MBL bind the serine protease MASP-2, which is then activated and cleaves C4 and C2 (17, 18). However, the role of LP in immunity against meningococci has not been clearly defined (16). In accordance with our previous study (28), our current SBA experiments with the anti-MASP-2 mAb showed no effect on meningococcal clearance in either pre- or post-vaccination sera, indicating that LP may not be essential.

In summary, our *in vitro* results with both serogroup B and non-B meningococci provide evidence for the potential safety advantages of AP inhibitors in comparison to C3 and C5 inhibitors, as long as treatment is accompanied by the vaccination of patients; however, this will finally have to be demonstrated in clinical trials. Our data confirmed that anti-fHbp antibodies play a key role in eliciting SBA against MenB isolates expressing a matching fHbp allele and revealed that complement activation by these protein-specific antibodies is remarkably resilient against alternative pathway inhibition. It cannot be ruled out that individuals treated with AP inhibitors may become more susceptible to invasive meningococcal disease when they are colonized by strains expressing only low levels of the target antigen or when antibody titers have declined.

## Data availability statement

The original contributions presented in the study are included in the article/**Supplementary material**. Further inquiries can be directed to the corresponding author.

## Ethics statement

The studies involving human participants were reviewed and approved by Ethical Committee of Northwest and Central Switzerland (Ethikkommission Nordwest- und Zentralschweiz (EKNZ), Studie 2018-02341). The patients/participants provided their written informed consent to participate in this study.

## Author contributions

EI and LM contributed to the study conceptualization, data acquisition, analysis, and data interpretation and drafted the manuscript. GP contributed to the study conceptualization and design, data analysis, and interpretation and revised the manuscript. AS, CT, NZ, TH, and MK contributed to the study conceptualization and design and revised the manuscript. All authors contributed to the article and approved the submitted version.
