# Supplementary material for: In vaccinated individuals serum bactericidal activity against B meningococci is abrogated by C5 inhibition but not by inhibition of the alternative complement pathway
Source: Front Immunol. 2023 Jun 29;14:1180833. doi: 10.3389/fimmu.2023.1180833 (PMC10349132; doi:10.3389/fimmu.2023.1180833)
Supplement: Supplementary file 1 [file DataSheet_1.pdf]

Supplementary Material

In vaccinated individuals serum bactericidal activity against B meningococci is abrogated by C5 inhibition but not by inhibition of the alternative complement pathway

Emma Ispasanie, Lukas Muri, Marc Schmid, Anna Schubart, Christine Thorburn, Natasa Zamurovic, Thomas Holbro, Michael Kammüller, and Gerd Pluschke*

*** Correspondence:** Gerd Pluschke: Gerd.Pluschke@swisstph.ch

1 Supplementary Data

2 Supplementary Figures and Tables

Supplementary Table 1. Summary of the average SBA titers of the human serum samples of subjects vaccinated with the MenB-4C vaccine Bexsero. SBA titers are the reciprocal dilutions of serum that result in 50% killing of the bacteria after 60 minutes incubation. A lack of SBA is indicated with the number 2 to indicate SBA titers below detection limit. The lowest serum dilution tested was 1:5. Abbreviations: pre vaccination serum sample (Pre), 2 weeks post booster (wPB), 2 months post-booster (mPB), 6 months post-booster (6mPB), 2 months post-dose 1 (mPD 1), 2 weeks post-dose 2 (wPD 2) and 6 months post-dose 2 (6mPD 2).

	**Subjects**	2				3				11				12			
	**Serum sample**	Pre	w PB	mPB	6mPB	Pre	mPD 1	wPD 2	6mPD 2	Pre	mPD 1	wPD 2	6mPD 2	Pre	mPD 1	wPD 2	6mPD 2
Mean SBA titer	MC58	6	26	27	21	2	44	41	21	2	12	25	6	11	99	64	48
	6105	2	26	16	2	2	68	98	20	2	3	46	2	2	24	27	7
	4263	2	2	2	2	2	2	2	2.5	2	10	2	8	22	19	22	2

2.1 Supplementary Figures

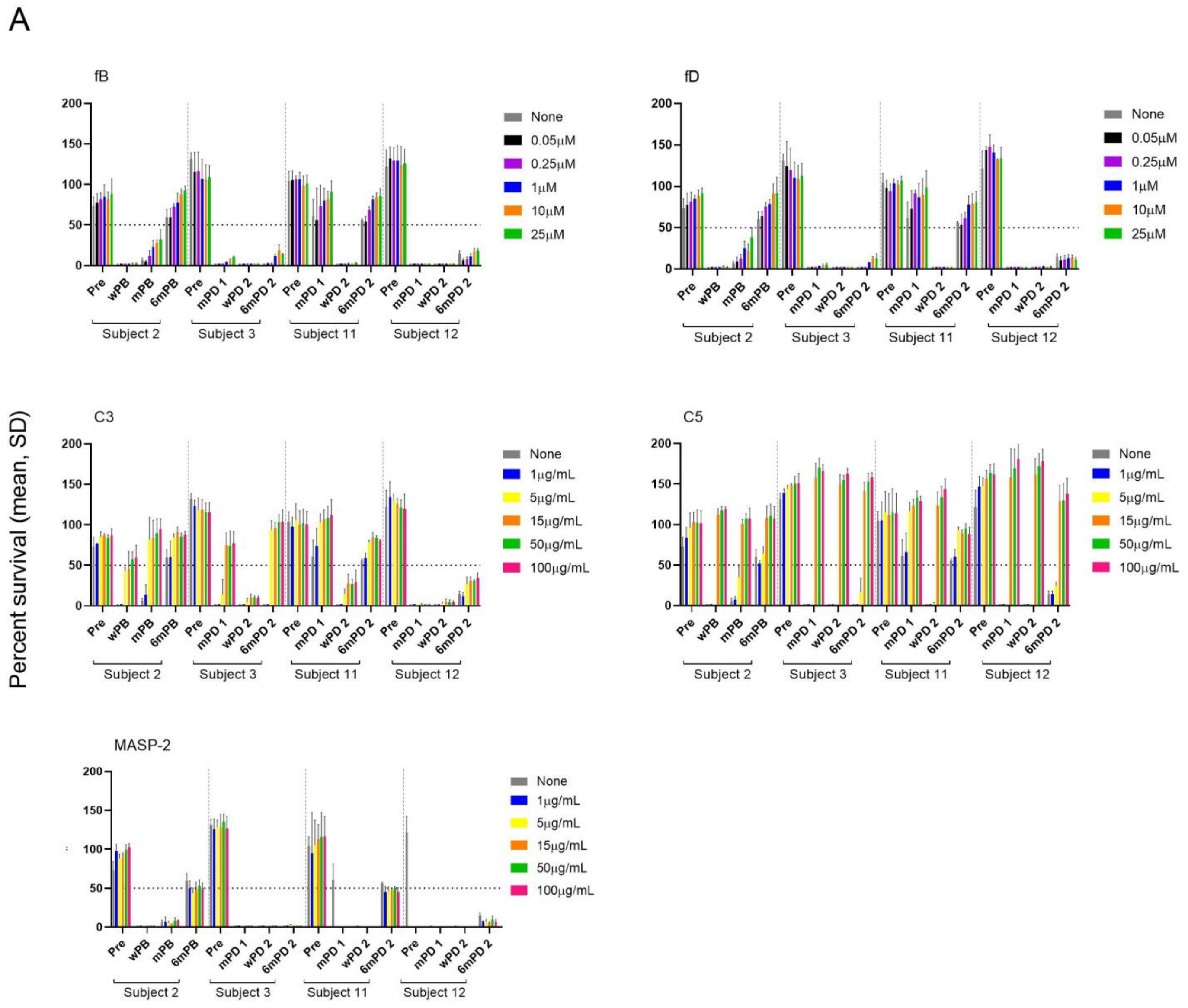

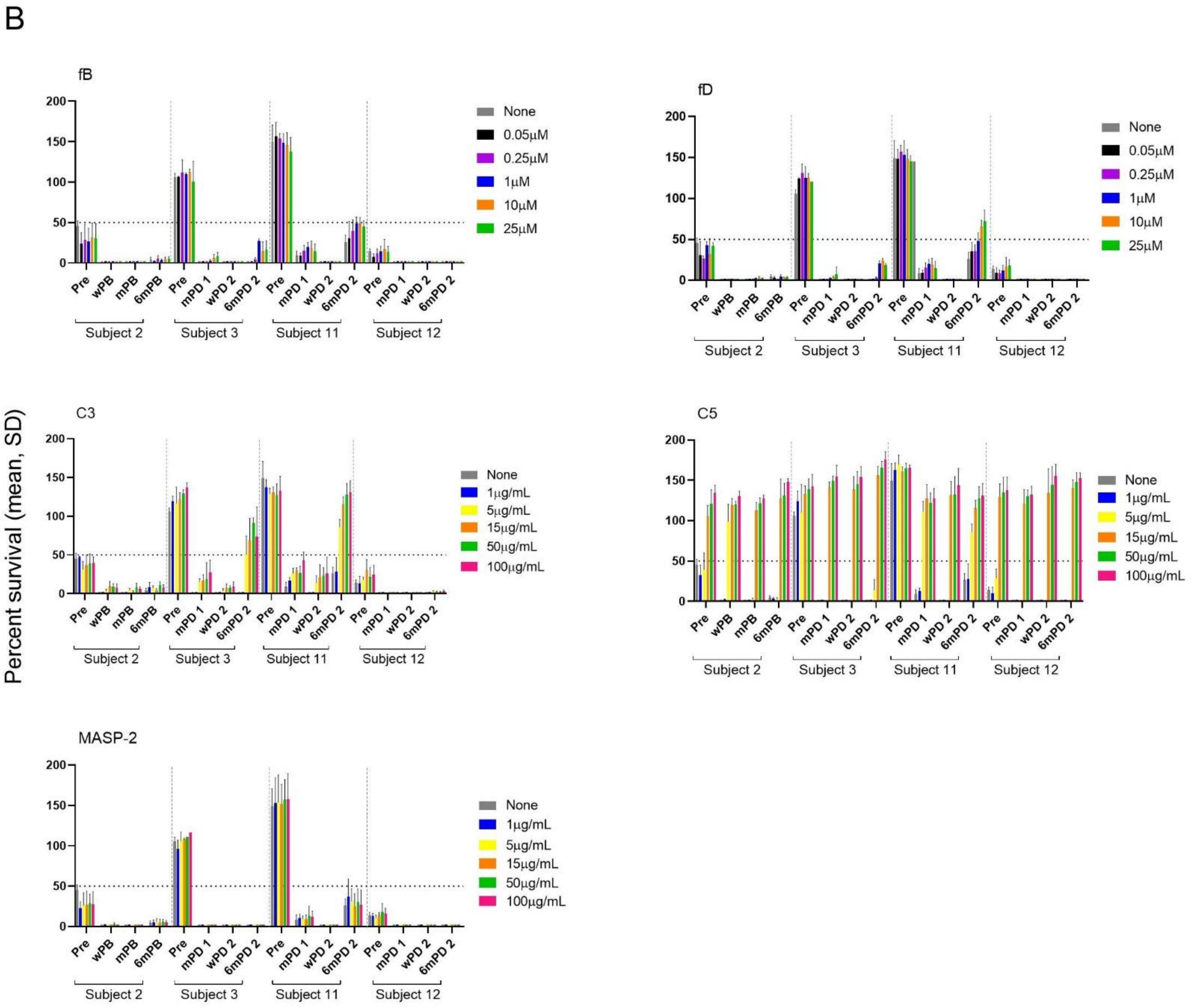
B
fB
fD
C3
C5
MASP-2
Percent survival (mean, SD)
None
0.05μM
0.25μM
1μM
10μM
25μM
1μg/mL
5μg/mL
15μg/mL
50μg/mL
100μg/mL
Pre
wPB
mPB
6mPB
mPD 1
wPD 2
6mPD 2
Subject 2
Subject 3
Subject 11
Subject 12
0
50
100
150
200

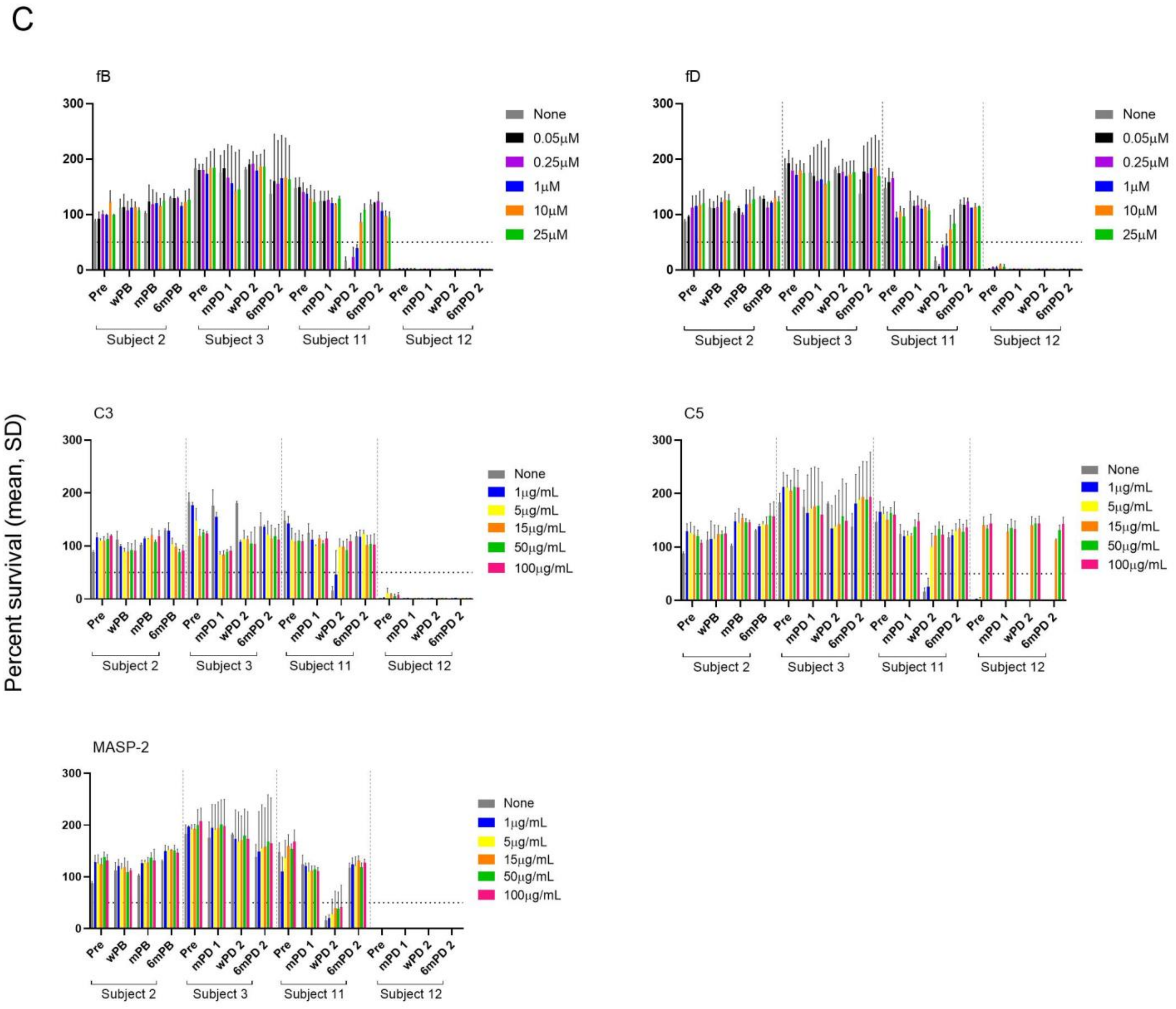

Supplementary Figure 1. The effect of complement inhibitors on the survival of serogroup B case isolates in pre- and post- 4CMenB vaccination sera. All subjects were vaccinated with the protein vaccine 4CMenB. All sera samples for each subject were diluted 1:5 and assayed with the internal complement and against meningococcal B case isolates 6105 (**A**), MC58 (**B**) and 4263 (**C**). The horizontal dotted line represents 50% killing of bacteria after 60 minutes incubation with different concentrations of factor B (fB) inhibitor iptacopan, factor D (fD) inhibitor CMS487, C3 inhibitor (C3) CP-40, anti-C5 monoclonal antibody (mAb; C5) tesidolumab and anti-MASP-2 mAb (MASP-2) narsoplimab. For each graph, grey bars are test sera without added inhibitors. Data for each inhibitor are triplicate technical replicates. SD: standard deviation of mean. Abbreviations: pre vaccination serum sample (Pre), 2 weeks post booster (wPB), 2 months post-booster (mPB), 6 months post-booster (6mPB), 2 months post-dose 1 (mPD 1), 2 weeks post-dose 2 (wPD 2) and 6 months post-dose 2 (6mPD 2).

Supplementary Figure 1. The figure legends are required to have the same font as the main text, 12 point normal Times New Roman, single spaced. Please use a single paragraph for each legend and prepare the figures keeping in mind the PDF layout.
